# The Competition of Yin and Yang: Exploring the Role of Wild-Type and Mutant p53 in Tumor Progression

**DOI:** 10.3390/biomedicines11041192

**Published:** 2023-04-17

**Authors:** Bi-He Cai, Yu-Te Sung, Chia-Chi Chen, Jei-Fu Shaw, I-Lun Hsin

**Affiliations:** 1 School of Medicine, I-Shou University, Kaohsiung City 82445, Taiwan; 2 Department of Plastic Surgery, E-Da Hospital, I-Shou University, Kaohsiung City 82445, Taiwan; 3 Department of Physical Therapy, I-Shou University, Kaohsiung City 82445, Taiwan; 4 School of Chinese Medicine for Post Baccalaureate, I-Shou University, Kaohsiung City 82445, Taiwan; 5 Department of Pathology, E-Da Hospital, I-Shou University, Kaohsiung City 82445, Taiwan; 6 Department of Biological Science and Technology, I-Shou University, Kaohsiung City 82445, Taiwan; 7 Institute of Medicine, Chung Shan Medical University, Taichung City 40201, Taiwan

The protein p53 is a well-known tumor suppressor that plays a crucial role in preventing cancer development. In Chinese philosophy, the concept of Yin and Yang is used to describe inseparable opposites, where Yin represents the negative or inhibitory force, and Yang represents the positive or promoting force. In this context, the wild type of p53 can be considered Yin, as it acts as a negative regulator of cancer progression, while most p53 mutations can be considered Yang, as they promote cancer progression.

Some p53 mutants, such as p53 S46F and S121F, exhibit higher transactivation activity than wild type, which can be considered as a super Yin of p53 [1,2,3]. Double mutations of p53 in mice, such as p53 F53Q and F54S, have been shown to enhance transactivation function and tumor suppression capacity, which can be considered as a super Yin of p53 [4]. The p53 protein forms a tetramer with four subunits, which are responsible for its fully transactivation function (Figure 1A) [5,6]. If we consider only the effect of mutated p53 on blocking wild type p53 activity, it takes three mutated p53 molecules within the tetramer to block the whole p53 tetramer transactivation activity (Figure 1B) [7]. In contrast, only one molecule of ΔNp53 isoform within the tetramer can break down the p53 tetramer transactivation activity (Figure 1C) [7]. Recent studies have revealed that some p53 mutants not only lose their transactivation function, but also gain oncogenic functions. One such function is their ability to become prion-like aggregation proteins that can pull down other tumor suppressor genes [8,9,10,11].

It has been known that some mutated p53 can appear in the cytosol [12], and cytosolic mutated p53 can block autophagy [13]. However, autophagy can also promote the degradation of mutated p53 [14,15]. The major aggregative proteins detected by the A11 antibody, which is specific to staining aggregative proteins, were found to co-localize with the signal detected by the p53 antibody within the nucleus of cells with p53 R280K mutation [9]. Previously, our research has shown that most p53 R175H molecules co-localize with thioflavin T (a stain reagent used to detect aggregative proteins) in the nucleus. However, some of these co-localized signals also appear in the cytosol [16]. A recent study reported that the presence of cytoplasmic mutant p53 aggregates is associated with poor prognosis in patients with high-grade serous ovarian carcinoma [17]. Therefore, cytosolic aggregated mutated p53 may be considered a super Yang molecule that promotes cancer progression. Some p53 mutants gain oncogenic functions by activating the PI3K-AKT pathway [18,19], which in turn inhibits autophagy [20,21]. Cytosolic aggregated mutated p53 may have an even greater effect in blocking autophagy through AKT or other oncogenic pathways. Moreover, autophagy may find it more difficult to clear aggregated mutated p53 in the cytosol. Additionally, AKT can phosphorylate MDM2, leading to the degradation of wild type p53 [22]. Functional members of the p53 family, such as p53, p63, and p73, can activate autophagy-related genes (ATGs) such as α2 and γ subunits of AMPK [23,24]. AMPK is a positive regulator of autophagy [25] and can also phosphorylate p53 family members to enhance their anticancer function [26,27]. p53, p63, and p73 can activate PUMA, which induces cell apoptosis [28,29,30]. However, the PI3K-AKT pathway can suppress PUMA expression [31,32], and as shown in Figure 2, mutant p53 can prevent cell apoptosis through the PI3K-AKT-PUMA pathway. We previously summarized the activators of p63 or p73 in p53 mutant cancers [33], and it is possible that the super Yang effect of cytosolic aggregated mutated p53 may confer higher drug resistance, making it harder to induce cancer cell apoptosis. Identifying drugs that can remove the super Yang effect of cytosolic aggregated mutated p53 or introducing/reactivating super Yin p53 family members to activate autophagy for cleaning mutated p53 are key issues in developing anticancer therapies.

## Figures and Tables

**Figure 1:**
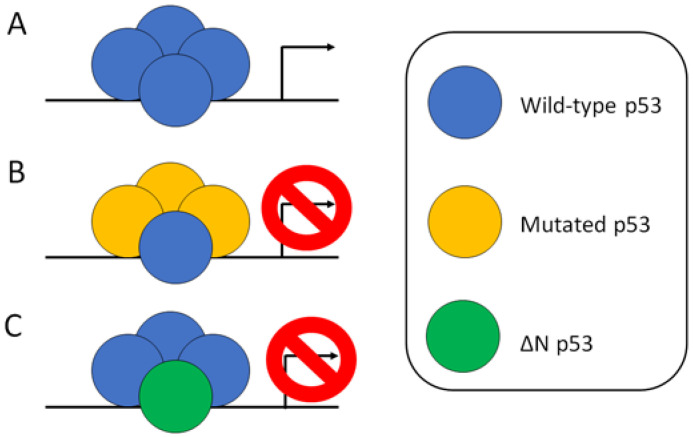
Effects of mutant p53 and ΔNp53 on the transactivity of wild type p53. (**A**) Wild type p53 binds to DNA response elements as a tetramer and activates the transcription of its target genes. (**B**) Mutant p53 can fully impair the transcriptional activity of wild-type p53 by forming a heterotetramer consisting of three mutant p53 subunits and one wild-type p53 subunit. (**C**) ΔNp53 is an isoform of p53 that lacks the transactivation domain. When one ΔNp53 subunit forms a heterotetramer with three wild type p53 subunits, the transcriptional activity of wild type p53 is completely abolished.

**Figure 2:**
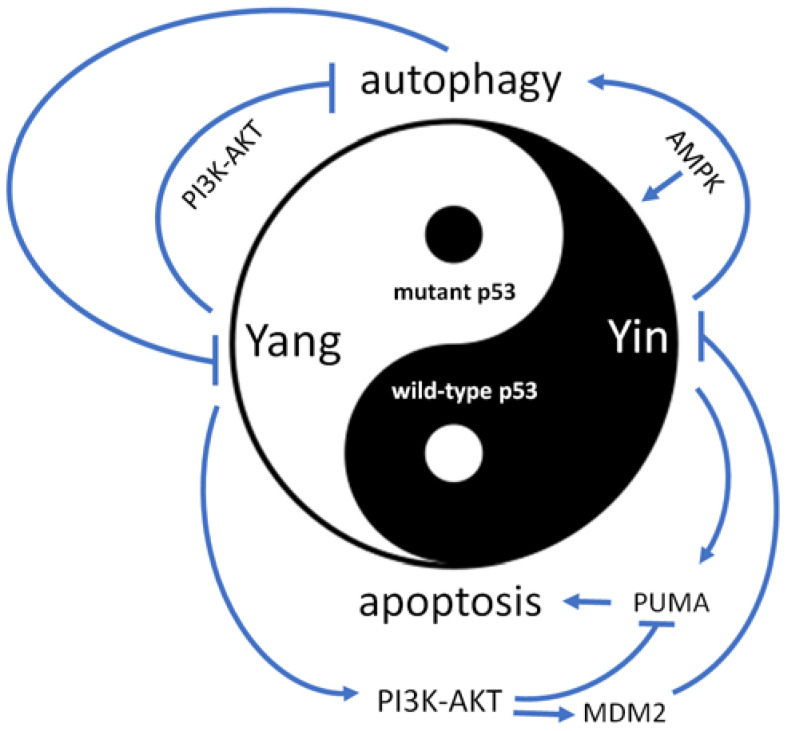
The competition between wild type and mutant p53 plays a crucial role in tumor progression. While wild type p53 acts as a negative factor (Yin), mutant p53 serves as a positive factor (Yang) in cancer development. Mutant p53 can activate the PI3K-AKT signaling pathway, which induces the MDM2-mediated degradation of wild type p53 and inhibits autophagy. On the other hand, wild type p53 can directly activate AMPK, which in turn activates autophagy and up-regulates p53′s anticancer function. Autophagy also plays a role in decreasing the expression of mutant p53. Wild type p53 can activate the pro-apoptotic protein PUMA, while the PI3K-AKT pathway can inhibit its expression. Mutant p53 can thus prevent cell apoptosis by activating the PI3K-AKT pathway.
